# Proton-Conducting Composite of Poly(2,5-benzimidazole) and Cesium Dihydrogen Phosphate—The Emerging of Ultrahigh-Temperature Polymer-Electrolyte Membrane Fuel Cell (UT-PEMFC)

**DOI:** 10.3390/membranes16060203

**Published:** 2026-06-10

**Authors:** Kirill M. Skupov, Igor I. Ponomarev, Elizaveta S. Vtyurina, Alexey A. Bugerya, Olga M. Zhigalina, Yulia A. Volkova, Anna A. Lysova, Yuri A. Dobrovolsky

**Affiliations:** 1 A.N. Nesmeyanov Institute of Organoelement Compounds of Russian Academy of Sciences, 28 Vavilova St., Bld. 1, Moscow 119334, Russia; ves1809@yandex.ru (E.S.V.); bugerya.alexei@yandex.ru (A.A.B.); yvolk@ineos.ac.ru (Y.A.V.); 2 Hydrogen Energy Center LLC, 3 Akademika Semenova St., Bld. 3, Chernogolovka 142432, Russia; dobr62@mail.ru; 3 Department of Materials Science, Bauman Moscow State Technical University, 5 2nd Baumanskaya St., Moscow 105005, Russia; zhigal@crys.ras.ru; 4 A.V. Shubnikov Institute of Crystallography Kurchatov Complex of Crystallography and Photonics National Research Centre “Kurchatov Institute”, 59 Leninsky Ave., Moscow 119333, Russia; 5 Kurnakov Institute of General and Inorganic Chemistry of the Russian Academy of Sciences, 31 Leninsky Ave., Moscow 119071, Russia; ailyina@yandex.ru

**Keywords:** proton-conducting membrane, cesium dihydrogen phosphate, poly(2,5-benzimidazole), ABPBI, polymer composite membrane, PEMFC, polymer-electrolyte membrane fuel cell, UT-PEMFC

## Abstract

Expansion of the operational temperature range for polymer-electrolyte membrane fuel cells (PEMFCs) above 200 °C significantly reduces hydrogen purification requirements. Here, we report a hybrid composite of poly(2,5-benzimidazole) (ABPBI) and CsH_2_PO_4_, doped with H_3_PO_4_, as a PEM for PEMFC operation at >200 °C up to 250 °C and beyond. The optimal ratio of ABPBI repeating units to CsH_2_PO_4_ is 1:1 (mol/mol). Materials are extensively characterized by elemental analysis, scanning electron microscopy, HAADF STEM, elemental mapping, electrochemical impedance spectroscopy, proton conductivity, mechanical testing, and Fourier transform infrared spectroscopy. It is suggested that PEMFCs with the extended operational temperature range (>220 °C) might be categorized as ultrahigh-temperature polymer-electrolyte membrane fuel cells (UT-PEMFCs).

## 1. Introduction

H_2_/air polymer-electrolyte membrane fuel cells (PEMFCs), based on a polymer proton-conducting membrane (PCM) [1,2], are promising sources in demand for electrical power that meet all requirements for the fourth energy transition towards carbon-free energy generation. With their unique power values and high efficiency levels, these products have already found a diverse range of applications, from portable energy sources to stationary setups with a power of hundreds of kW. H_2_-fueled cars and buses equipped with fuel cells (FCs) are commercially available [3]. FCs capable of operating heavy cargo vehicles, river boats and even aircrafts are at the testing stage [4]. The PEMFCs include a group of low temperature ones (LT-PEMFCs), which usually operate with a Nafion^®^ membrane at <100 °C [5], as well as intermediate-temperature PEMFCs (IT-PEMFCs), which operate at around 100–140 °C, with additional humidification only [6]. Additionally, high-temperature polymer-electrolyte membrane fuel cells (HT-PEMFCs) operate with a polybenzimidazole/phosphoric acid (PBI/PA) polymer-electrolyte complex as a membrane which is mostly based on either poly [2,2′-*m*-(phenylene)-5,5′-bibenzimidazole] (*m*-PBI) [1,7,8,9,10,11] or poly(2,5-benzimidazole) (ABPBI) [12,13,14,15], and their operational temperature range is typically limited to 200–220 °C without any additional humidification. ABPBI-based membranes possess a number of advantages over *m*-PBI, such as higher PA uptake and lower cost of production [12]. The extremely high glass transition temperature of pure ABPBI materials (T_g_ > 500 °C) provides the required strength. The maximum power for a pristine (non-enhanced) ABPBI membrane is about 0.2 mW/cm^2^ at 160–180 °C [15,16]. However, a disadvantage is their inability to apply to ABPBI/PA complexes as a PCM at temperatures above 200 °C [12] due to their dissolution (or creeping) in PA. Extending the operating temperature range of PEMFC while maintaining membrane stability and high proton conductivity becomes a significant challenge. It would ultimately enhance the FC’s specific power output and enable the FC to operate with H_2_ contaminated with higher levels of impurities, such as carbon monoxide. Cesium dihydrogen phosphate (CDP), CsH_2_PO_4_, which has been previously shown to be suitable as a PCM in FCs, has been found to have a low power output due to ohmic losses [17,18]. It has been suggested that a combination of CDP with a polymer used for membrane production, such as polyvinyl butyral [19], polytetrafluoroethylene [20], polyvinylidene fluoride [21], or epoxy resins [22], can result in improved mechanical properties and thinner membranes. In turn, it would lead to lower ohmic losses; however, proton conductivity is still too low. The combination of ABPBI and CDP might significantly enhance the mechanical properties of a composite membrane while maintaining high proton conductivity. Based on our previous research experience with HT-PEMFCs [23,24,25,26,27], we have recently presented very preliminary results on the proton conductivity of fragile membranes based on ABPBI and CDP doped with PA up to 90 °C [28]. As previously stated, the use of ABPBI as a polymer binder for CDP is justified due to its extremely high thermal and heat resistance, high mechanical strength in films and fibers and extremely high fire resistance, as well as its ability to form stable complexes with PA, which possesses high proton conductivity at 150–200 °C. ABPBI films doped with 3–5 PA molecules per polymer repeating unit are successfully tested in HT-PEMFCs and show properties which are competitive with commercially available membrane electrode assemblies (MEAs) such as Celtec-P1000 and Dapozol which used membranes based on *m*-PBI [29]. In most publications on ABPBI, in addition to the unique properties mentioned above, its affordability and potentially low production cost are often noted, because industrial and inexpensive 3,4-diamino benzoic acid is used in its synthesis. However, for the production of the most commonly used and commercially available *m*-PBI, an expensive and carcinogenic monomer, 3,3-diaminobenzidine, is required. The only significant challenge related to the industrial production of ABPBI and its conversion into films and fibers is the fact that it is soluble only in strong acids. In our previous studies, we have shown that even a high-molecular-weight ABPBI (M_w_~100 kg/mol) is well soluble in a complex organic solvent composed of DMSO, MeOH and KOH [30]. Using these 5–10% solutions, strong films and fibers can be obtained without the need for aggressive acidic solvents such as sulfuric acid, methane sulfonic acid or mixtures of trifluoroacetic acid (TFA) with formic acid (FA). Previously, we have employed ABPBI as a polymer binder for CDP. It has been possible to produce a membrane by dissolving crystalline CDP and ABPBI in a mixture of TFA and FA, with further addition of PA, in order to dope ABPBI with 1–5 PA molecules per ABPBI repeating unit. The resulting proton-conducting membranes have proven to be highly fragile, but very preliminary results suggest they have still been suitable for certain testing in HT-PEMFCs up to 250 °C [28]. Better film properties in DMSO-based solvents are likely related to a different mechanism of ABPBI dissolution in DMSO-based solvents and TFA/FA. Due to a superbasicity effect in the DMSO/CsOH/MeOH solvent system (a thermodynamically better solvent than TFA/FA), which is associated with the formation of the methoxide ion (MeO¯), the imidazole ring of ABPBI actively deprotonates and forms polyanions, ensuring complete disentanglement of chains, which, in turn, improves its solubility and leads to higher ABPBI concentration during evaporation. High polymer concentration is crucial for the formation of dense, continuous, and defect-free films. In the case of more rapidly evaporating TFA/FA solvent (due to much lower T_b_ of TFA compared with DMSO), polymer shrinkage may be related to a lower ABPBI concentration, leading to a more fragile and porous (and potentially more defective) film. Despite protonation of the imidazole rings, the TFA/FA solvent may not provide the required level of chain de-entanglement prior to film formation, due to relatively weaker interactions with the solvent, resulting in “aggregates” during evaporation and less uniform film morphology. CDP distribution was also expected to be less uniform in the case of a TFA/FA solvent.

According to [31], a similar cesium hydrogen phosphate (CHP)/PBI MEA based on a *m*-PBI/CsH_5_(PO_4_)_2_ membrane reached 120 mW cm^−2^ at 200 °C in H_2_/O_2_ operation, speculating that under operation conditions, CsH_5_(PO_4_)_2_ is in a molten state. In order to pursue a promising direction, it has been decided to adopt an innovative approach, which involves preparing ABPBI solutions using the DMSO-MeOH-KOH complex organic solvent [30], with KOH replaced by CsOH. Based on these results [30], ultimately, we concluded that CDP could be formed in situ from CsOH and PA in a DMSO-MeOH-CsOH solvent, resulting in improved film quality. As a result, the film’s quality would be improved due to lower shrinkage, which is likely related to slower solvent evaporation. From these 5–10 wt.% solutions, strong and flexible films were obtained, which were subsequently been treated with PA in order to convert all CsOH into CDP and to dope the ABPBI with three PA molecules per polymer unit.

If the obtained ABPBI/CDP composite can withstand higher temperatures, the operating temperature range of PEMFCs would be extended up to 250 °C and beyond, resulting in the emergence of a new type of ultrahigh-temperature polymer-electrolyte membrane fuel cell (UT-PEMFC) with the possibility of operating at >220 °C. To date, there are a very limited number of references, for example [32,33], for PEMFCs that might be categorized as UT-PEMFCs. The aim of this study is to extend the operating temperature range of PEMFCs beyond 200–220 °C by developing a novel composite proton-conducting membrane based on ABPBI and CDP.

## 2. Materials and Methods

### 2.1. Materials

Phosphoric acid (PA), polyphosphoric acid (PPA), 3,4-diaminobenzoic acid (DABA), cesium hydroxide monohydrate (CsOH·H_2_O), dimethyl sulfoxide (DMSO), and methanol are Sigma-Aldrich (Merck, Darmstadt, Germany) products and are used as received. Crystals of cesium dihydrogen phosphate (CDP), CsH_2_PO_4_, are grown by slow isothermal evaporation of PA (analytical grade) from aqueous solutions and Cs_2_CO_3_ (analytical grade), taken in an equimolar ratio at room temperature. X-ray diffraction (XRD) analysis of the obtained CsH_2_PO_4_ (P21/m) fully corresponds to the crystallographic database (35–746).

### 2.2. Methods

#### 2.2.1. ABPBI Preparation

**Figure 1:**
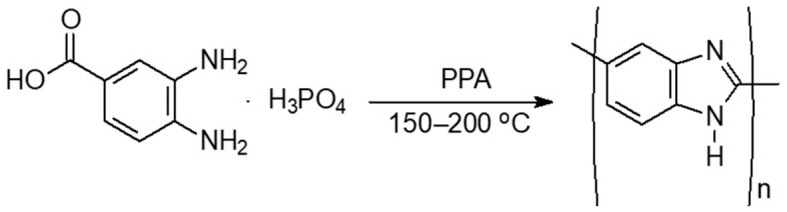
ABPBI synthesis from DABA in PPA.

ABPBI was obtained according to a general procedure [30] from DABA monophosphate in PPA (Figure 1).

Briefly, 75 g of PPA (84%) is introduced to a flask with a capillary for argon supply and is stirred at 120 °C under an argon flow. DABA monophosphate (25 g, 0.1 mol) is added portionwise under stirring. Stirring is continued for 2 h until a 25% homogeneous solution is obtained. One part of the reaction mixture is poured into Teflon cups and heated to 120 °C (in a muffle furnace). Finally, the resulting reaction solutions in PPA are heated to 180–200 °C at a rate of 1 K/min and kept for 20 h. To isolate the polymers, the solutions are treated with hot deionized water, ground, and treated with 5% aq. ammonia at ~20 °C for 3 days. The product is extracted with methanol using a Soxhlet apparatus for removal of low-molecular-weight impurities. Then, the obtained polymer is dried under vacuum (~10^−2^ mbar) for at least 2 h at 150 °C, providing a typical ABPBI polymer with intrinsic viscosity [η] of 4.0 dL/g (H_2_SO_4_, 25 °C, M_w_ corresponds to ~42 kg/mol). The yield of the product is ~99%. Subsequently, the obtained ABPBI polymer was used to produce proton-conducting membranes.

#### 2.2.2. Preparation of the Composite Membranes (ABPBI_PRU_·3PA/nCDP)

The dissolution of ABPBI is performed in sealed vials. To obtain three composites with different ratios of ABPBI polymer repeating units (ABPBI_PRU_) to CDP, ABPBI (1.16 g, 0.01 mol) and CsOH·H_2_O (1.344 g, 0.008 mol) for ABPBI_PRU_/CDP 1:0.8; ABPBI (1.16 g, 0.01 mol) and CsOH·H_2_O (1.680 g, 0.01 mol) for ABPBI_PRU_/CDP 1:1; and ABPBI (1.16 g, 0.01 mol) and CsOH·H_2_O (2.015 g, 0.012 mol) for ABPBI_PRU_/CDP 1:1.2 are heated with stirring in a mixture of DMSO (13 g) with MeOH (7 g) at 100 °C until a viscous solution is formed. The ABPBI/CsOH membranes are cast using an automatic film coater MemCast (Porometer NV, Nazareth, Belgium) with casting knives, offering a wet coating thickness of 500 µm on a metal substrate at 30 °C (Figure S1).

The membranes are dried for 24 h on a metal substrate. When the solvent is evaporated, the entire film material is cut into squares (5 × 5 cm) and immersed in 20 mL of PA aqueous solution to reach a doping level of 3 PA molecules per 1 polymer repeating unit of ABPBI and convert CsOH to CsH_2_PO_4_. The PA aqueous solutions contain 3.72 g of PA for the composite with the ABPBI_PRU_/CDP ratio of 1:0.8, 3.92 g of PA for the composite with the ABPBI_PRU_/CDP ratio of 1:1, and 4.12 g of PA for the composite with the ABPBI_PRU_/CDP ratio of 1:1.2. The composite films are kept in Teflon cups in PA solutions for three days. During this time, the lower membranes were periodically moved to the top to ensure a uniform doping process. After three days of doping, the cups were placed in an oven with circulating air at 60 °C for 24 h to evaporate the water. As a result, three membranes of ABPBI_PRU_·3H_3_PO_4_/nCsH_2_PO_4_ (where n is 0.8, 1.0 and 1.2, correspondingly) with a thickness of 90 µm (membrane samples **1**, **2** and **3**, for the ABPBI_PRU_/CDP ratios of 1:0.8, 1:1, 1:1.2, respectively) are obtained for testing (Figure S2).

The membrane elemental analysis data, as well as quantitative values for the components taken for the formation of composite solutions in 20 g of DMSO/MeOH (65:35 *w*/*w*) for ABPBI/CsOH and ABPBI_PRU_·3H_3_PO_4_/nCsH_2_PO_4_ for different ABPBI_PRU_/CsH_2_PO_4_ ratios, are provided in Table 1.

#### 2.2.3. Membrane Proton Conductivity Measurements

Through-plane proton conductivity (S/cm) measurements of the membranes are performed by electrochemical impedance spectroscopy on an Elins Z500-PRO impedance meter (Chernogolovka, Russia) in a frequency range of 10–2 × 10^6^ Hz in potentiostatic mode with a sinusoidal excitation voltage of 100 mV in a two-electrode cell, with graphite electrodes in a range of 25–270 °C and a step of 10–15 K. The proton conductivity value is determined by extrapolation of semicircles of the conductivity volume component to the axis of active resistances.

#### 2.2.4. Membrane Electrode Assembly Fabrication

Celtec^®^-P Series 1000 cathodes and anodes (BASF Fuel Cell, Frankfurt am Main, Germany) [34] are assembled with proton-conducting membranes ABPBI_PRU_·3H_3_PO_4_/nCsH_2_PO_4_ (**1**, **2** and **3**), with different ABPBI_PRU_/CDP (mol/mol) ratios of 1:0.8; 1:1; and 1:1.2, resulting in membrane electrode assemblies (MEAs) labeled **MEA 1**, **MEA 2** and **MEA 3**, respectively. For testing, the MEAs are placed between two graphite flow field plates in standard Arbin Instruments testing cells (College Station, TX, USA); the MEA working area is 5 cm^2^.

#### 2.2.5. Fuel Cell Testing

The fuel cells are operated in the range 160–250 °C. The polarization curves are recorded using an Elins P-200X Potentiostat (Electrochemical Instruments, Chernogolovka, Russia) at 160–250 °C and ambient pressure, with a voltage scanning rate of 5 mV s^−1^. The gases are supplied without additional humidification. The anode is supplied with hydrogen at a rate of 100 mL/min and the cathode is supplied with air at a rate of 800 mL/min.

#### 2.2.6. Electrochemical Impedance Spectroscopy

Membrane resistance (mΩ cm^2^) is measured by in operando electrochemical impedance spectroscopy (EIS) during the operation of membrane electrode assembly at 0.4 A cm^−2^ from the corresponding impedance Nyquist plot in the high-frequency region on a SmartStat PS-80 potentiostat–galvanostat (Electrochemical Instruments, Chernogolovka, Russia), with a sinusoidal current, amplitude of 50 mA and frequency range of 50 kHz–0.1 Hz.

#### 2.2.7. Hydrogen Crossover Current Measurements

For measurements of hydrogen crossover current through the membrane, the method of linear sweep voltammetry (LSV) [1] is applied. The cathode is supplied with argon at 50 mL/min; the anode is supplied with hydrogen at 100 mL/min. The gases are supplied at an ambient pressure until the open-circuit voltage reaches a pseudo steady-state value of ~120 mV. The voltage is increased slowly at a rate of 1 mV s^−1^ to 500 mV. The resulting current of hydrogen oxidation is recorded. Alternatively, the H_2_ crossover value was obtained at 500 mV after 30 min of current recording.

#### 2.2.8. Tensile Testing

The mechanical tests of proton-conducting membranes are carried out on the 2166 R-5 tensile strength testing machine (Tochpribor, Ivanovo, Russia) at room temperature and ambient pressure in tension mode with the crosshead speed of 10^−4^ m s^−1^. The standard sample (20 × 1.5 mm) is used for the tests. The mechanical characteristics (tensile strength σ, elongation at break ε and Young’s modulus E) are found from the stress–strain curves. The beginning linear part of the curve is used to find the Young’s modulus. Five experiments are conducted.

#### 2.2.9. Elemental Analysis

An Elementar vario micro cube C,H,N-analyzer (now Elementar Analysensysteme, Langenselbold, Germany) equipped with a thermal desorption column is applied to determine the elemental composition. For P determination, a Cary 100 Scan UV–Vis spectrophotometer (Agilent Technologies, Santa Clara, CA, United States) is used after sample decomposition with concentrated sulfuric acid to analyze resulting heteropoly acids using spectrophotometry, according to the Kjeldahl method. For Cs determination, the X-ray fluorescence (XRF) method is employed with a Spectroscan Max GVM (Spectron, St. Petersburg, Russia).

#### 2.2.10. Electron Microscopy

Scanning transmission electron microscopy with a high-angle annular dark-field detector (HAADF STEM) and elemental mapping are performed using an FEI Tecnai Osiris system (FEI, Hillsboro, OR, United States) with accelerating voltage of 200 kV, equipped with a special SuperX EDS system which includes four silicon detectors to obtain chemical distribution maps. The scanning electron microscopy (SEM) images of ABPBI/CDP are obtained from FEI Scios (FEI, Hillsboro, OR, United States). The obtained electron microscope images are analyzed using EDAX APEX 2.0 TIA 16 (Siemens, Munich, Germany), JEMSJEMS software (EMS Java version 2004, P. Stadelmann JEMS, EPFL, https://www.jems-swiss.ch/, accessed on 24 May 2026, Lausanne, Switzerland),Esprit 2 (Bruker, Billerica, MA, USA) and DigitalMicrograph Gatan Microscopy Suite GMS 3 (Gatan, Pleasanton, CA, USA).

#### 2.2.11. Fourier Transform Infrared Spectroscopy

Fourier transform infrared spectroscopy (FTIR) of individual compounds, either in KBr pellets or as polymer films, is performed in absorbance mode using an InfraRed Bruker Tensor 37 FTIR spectrometer (Bruker Optics, Ettlingen, Germany) with a resolution of <0.6 cm^−1^ and spectral range of 7500–370 cm^−1^.

## 3. Results and Discussion

ABPBI was obtained from 3,4-diaminobenzoic acid (DABA) monophosphate in polyphosphoric acid (PPA) according to Figure 1. The polymers possess molecular weights of ~50 kg/mol, and the DMSO-MeOH-CsOH solutions of ABPBI are obtained. It should be noted that in order to achieve high-quality solutions of ABPBI in the presence of KOH, at least an equimolar ratio of ABPBI polymer repeating units (ABPBI_PRU_) to KOH is required for complete protonation of the -NH- groups in ABPBI and its transfer to solution. Since CsOH is a stronger alkali than KOH, it has been possible to obtain a solution of ABPBI with a smaller amount of CsOH, specifically 0.8 mol per 1 mol of ABPBI_PRU_. To study the effect of cesium concentration on the final proton-conducting composite material, solutions of ABPBI containing 0.8, 1.0 and 1.2 mol of CsOH per 1 mol of ABPBI_PRU_ were prepared. From these solutions, strong and elastic films formed (Figure S1). The films were treated with diluted PA in the amount necessary to convert all CsOH to CDP and dope ABPBI with three PA molecules per PRU, followed by the evaporation of water. A comparison of calculated and experimentally found elemental analysis data suggests near-complete reaction of CsOH.

**Figure 2:**
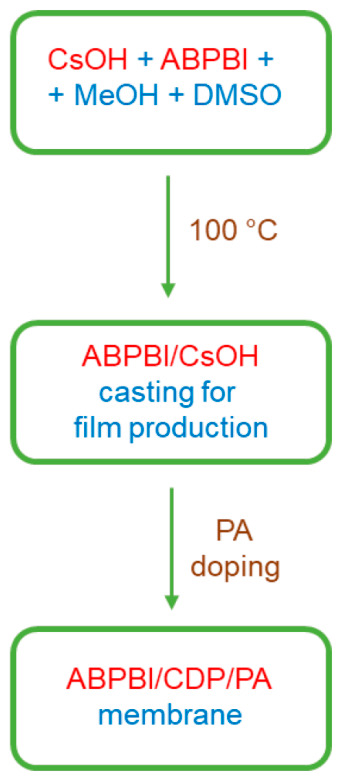
A general scheme of the ABPBI_PRU_·3H_3_PO_4_/nCsH_2_PO_4_ membrane obtaining process.

A general scheme of how we obtained the membrane is provided in Figure 2.

**Figure 3:**
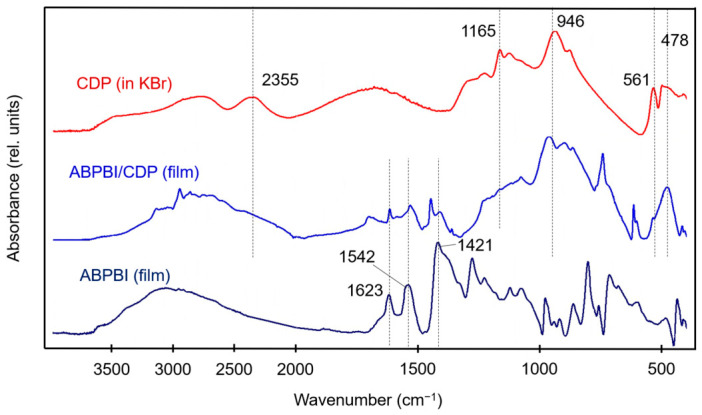
FTIR spectra for CDP in KBr, ABPBI_PRU_·3PA/CDP (**2**, film, ABPBI/CDP 1:1 mol/mol) and ABPBI (film).

The chemical compositions of ABPBI·3PA/CDP composites **1–3** (Figure S2), with ABPBI_PRU_/CDP molar ratios of 1/0.8; 1/1 and 1/1.2, respectively, are fully confirmed by data from elemental analysis (Table 1) and FT-IR spectroscopy (Figure 3). It becomes possible to work with an increased amount of CDP (compared with the previous study [28]) because it is formed in situ during ABPBI film formation from the thermodynamically good solvent DMSO-MeOH-CsOH.

**Figure 4:**
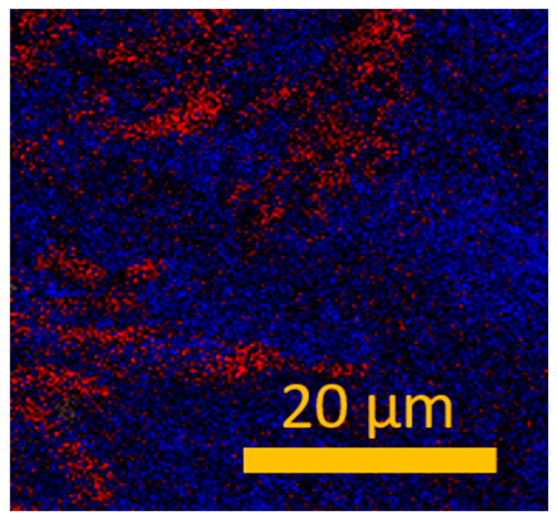
Elemental maps of Cs (red) and C (blue) from a HAADF STEM image for ABPBI_PRU_·3PA/CDP, membrane **2** (ABPBI/CDP 1:1 mol/mol).

A number of broad bands in the range 1500–2800 cm^−1^ corresponds to a strong network of CDP hydrogen bonds [19]. Bands at 800–1300 cm^−1^ correspond to various phosphate fragments, particularly, P-OH stretch and P-O bend vibrational modes; bands at 400–800 cm^−1^ belong to O-P-O bend and Cs-O stretch vibrational modes [35,36]. The band at 946 cm^−1^ is very strong and is not observed for ABPBI at all. The elemental analysis data, provided in the experimental part, clearly correspond to the composite composition. For the ABPBI spectrum, the characteristic peaks for the benzimidazole ring are observed at about 1421, 1542, and 1623 cm^−1^ [37]. The FTIR spectrum for its composite ABPBI_PRU_·3PA/CDP (1:1 mol/mol) is also provided; its spectrum clearly represents a superposition of the individual spectra of its constituent components. The mechanical characteristics of proton-conducting membrane **2** (tensile strength σ 40 ± 10 MPa; elongation at break ε 6 ± 3%; and Young’s modulus E 1440 ± 150 MPa) indicate the possibility to apply the material to FC MEAs. Figure S3 represents SEM images of ABPBI_PRU_·3H_3_PO_4_/CsH_2_PO_4_, membrane **2**, with a ABPBI_PRU_/CDP (mol/mol) ratio of 1:1. CDP is uniformly distributed within the membrane, forming large-size crystal-like particles. The elemental map confirms its uniform distribution (Figure 4 and Figure S4; Table S1).

In the case of the DMSO-based solvent, CDP particles are relatively small and more uniformly distributed compared with the case of films in the TFA/FA solvent [28], where the distribution of CDP particles was expected to be less uniform because of the reasons discussed before. In order to find the membrane resistance during FC operation, *in operando* EIS measurements are performed. Three fuel cells with membrane electrode assemblies (MEAs) **1–3** are assembled and designated **MEA 1**, **MEA 2** and **MEA 3**, respectively. A series of Nyquist plots obtained from EIS data for **MEA 1** at different temperatures under FC operating conditions is provided in Figure S5. The lowest resistance was observed at 200 °C, despite expectations that CDP could increase proton conductivity through transformation into a proton-conducting phase. As a result, the series of resistance values (R_t_) at different temperatures (t) can be represented as R_200_ (131.1 mΩ cm^2^) < R_220_ (143.7 mΩ cm^2^) < R_180_ (170.8 mΩ cm^2^) < R_240_ (180.3 mΩ cm^2^) < R_160_ (204.6 mΩ cm^2^). A very different situation is observed for **MEA 2** (Figure S6). In this case, an increase in temperature correlates with a decrease in resistance. The series of resistance values is R_250_ (84.1 mΩ cm^2^) < R_180_ (86.9 mΩ cm^2^) < R_160_ (103.6 mΩ cm^2^). However, the difference between 180 and 250 °C is not significant, which is due to proton transfer in a solid electrolyte with a lower amount of water at >240 °C. The EIS Nyquist plot data for **MEA 3** is presented in Figure S7. It can be seen that the resistance at 180 °C (179.9 mΩ cm^2^) is lower compared with the one at 160 °C (235.9 mΩ cm^2^). Such high resistance values can be explained by a higher amount of CDP solid electrolyte in the composite membrane.

**Figure 5:**
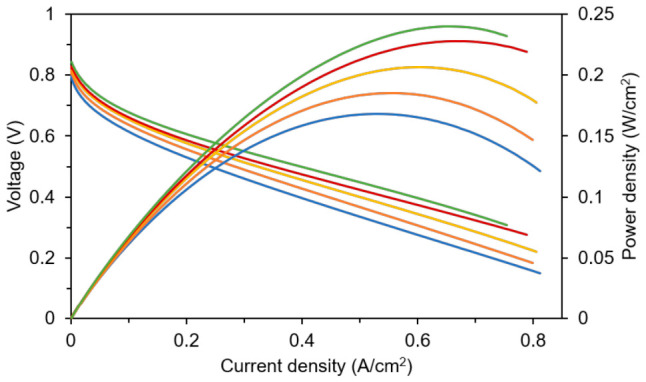
Polarization and power density curves for **MEA 2** at 160 °C (blue), 180 °C (orange), 200 °C (yellow), 220 °C (red) and 250 °C (green).

Polarization and power density curves for **MEA 1** taken at different temperatures are shown in Figure S8. At 220–240 °C, maximal values of power density are observed. In addition to the temperature effect, the increase may be related to the transfer of CDP to the proton-conducting phase. An increase in power values (P_t_) with increasing temperature (t) is observed: P_180_ (0.160 W/cm^2^) < P_200_ (0.163 W/cm^2^) < P_220_ (0.194 W/cm^2^) < P_240_ (0.199 W/cm^2^). The analysis of polarization and power density data leads to the conclusion that CDP, as part of the composite membrane, is able to enhance the performance of the MEA through its transition to the tetragonal phase. Polarization and power density curves for **MEA 2** are presented in Figure 5.

In this case, Figure 5 also shows an increase in power values (P_t_) with increasing temperature (t): P_160_ (0.16 W/cm^2^) < P_180_ (0.18 W/cm^2^) < P_200_ (0.20 W/cm^2^) < P_220_ (0.21 W/cm^2^) < P_250_ (0.24 W/cm^2^). The initial values could be explained by a limited calculated amount of PA in the MEA. After 2 weeks, the maximal power density value increases to 0.31 W/cm^2^ at 180 °C (Figure S9). However, the values after 24 h of operation are taken for comparison at different temperatures in order to correctly reveal the role of CDP. The MEA operation is stable for ~5 h at 250 °C and ~50 h at 200 °C. The membrane itself (ABPBI/CDP complex with PA) was treated at 250 °C for 8 h in air a few times to control its integrity. No changes are detected. A polarization curve at 250 °C starts to suffer from mass transfer loss problems at high current densities, which is probably due to the necessity of a more porous electrode (rather than a BASF electrode) and the elimination of a Teflon polymer from commercial electrodes for operation at this temperature. Polarization and power density curves for **MEA 3** are presented in Figure S10. The following order of power values (P_t_) with increasing temperature (t) is obtained: P_160_ (0.083 W/cm^2^) < P_180_ (0.102 W/cm^2^) < P_200_ (0.124 W/cm^2^). The value of P_160_ after 2 days is 0.102 W/cm^2^. These values are quite low, which indicates that the ABPBI_PRU_:CDP ratio of 1:1.2 is not the optimal one. It is clear from the presented data, that in terms of the resistance values and power density (Figures S11 and S12), the optimal ABPBI_PRU_/CDP ratio is found to be 1:1. The BASF electrodes do not create conditions for good mass transfer at >200 °C, indicating that alternative electrodes with a more highly developed porous structure may need to be considered in order to achieve optimal performance at these elevated temperatures. Due to the low mechanical stability of pristine ABPBI, its hydrogen crossover values are rarely reported in the literature. In our case, the hydrogen crossover through the membrane value at a constant potential of 500 mV is found to be 6.1 mA/cm^2^ for **MEA 2** equipped with membrane **2**, possessing a 1:1 ABPBI_PRU_/CDP (mol/mol) ratio. Alternatively, the linear sweep voltammetry (LSV) method is applied for **MEA 2** with membrane **2**, to assess hydrogen permeability through the membrane at 160 °C (Figure S13). The current density related to hydrogen crossover through the membrane with short-circuit correction (SCC) is found to be 1.5–1.8 mA/cm^2^, which is even lower than 4–5 mA/cm^2^ for *m*-PBI membranes [38] in commercial Celtec P-1000 MEA (BASF) [34]. Regardless, both values for ABPBI/CDP, with and without SCC, are lower than the hydrogen crossover of a standard ABPBI membrane, which has been reported to be 8 mA/cm^2^ at 150–175 °C [15]. These data indicate improved H_2_ barrier properties of CDP/ABPBI compared with a pristine ABPBI membrane. For MEAs with better performances (**MEA 1** and **MEA 2**), membranes **1** and **2** were further tested for through-plane proton conductivity over the temperature range 25–270 °C with an interval of 10–15 °C (Figure S14). It is found that the initial proton conductivity of the membranes is higher for the ABPBI membrane with an ABPBI_PRU_/CDP ratio of 1:0.8 (mol/mol). However, the increase in conductivity as a result of the phase transition is more pronounced for the ABPBI membrane with an ABPBI_PRU_/CDP ratio of 1:1 (mol/mol) and is observed between 180 and 230 °C. For sample **1**, the same effect is observed at 190–240 °C. Further, an increase in temperature results in slightly lower proton conductivity values, presumably due to the PA oligomerization reactions and the formation of CsH_5_(PO_4_)_2_. It is important to note that these results are obtained under dry conditions, with no water production, compared with *in operando* conditions. In addition to the 150–200 °C range, the obtained results suggest the possibility of using these membranes for UT-PEMFCs at 200–270 °C.

## 4. Conclusions

The performance of the ABPBI/CDP composite doped with PA as a PCM in PEMFCs at >220 °C up to 250 °C and beyond depends on the ABPBI/CDP ratio and allows for the expansion of the PEMFC operation temperature range. Pristine ABPBI is not able to tolerate high temperatures since they lead to the deterioration and severe dehydration of the membrane at > 200 °C. CDP is assumed to stabilize and enhance the ABPBI composite materials and help to retain water more effectively in the membrane. Proton transport is supposed to occur through transport channels along ABPBI/CDP phase boundaries and is related to protonated forms of ABPBI and CDP (Figure S15). Such PEMFCs might be categorized as emerging ultrahigh-temperature polymer-electrolyte membrane fuel cells (UT-PEMFCs) with a possibility to operate at >220 °C, which is a separate category in addition to LT-PEMFCs [39,40,41], IT-PEMFCs [42,43,44,45] and HT-PEMFCs [46,47].

Nevertheless, the currently used BASF electrodes are not able to provide the optimal conditions for mass transfer at >200 °C. Therefore, future directions are related to alternative electrodes with a more advanced porous structure and more stable electrocatalyst support, which have to be explored in order to achieve optimal performance at higher temperatures.

## Figures and Tables

**Table 1:** The membrane elemental analysis data and quantitative values for the components taken for formation of composite solutions to obtain of the ABPBI·3PA/CDP composite membranes.

Sample	Ratio (mol/mol) ABPBI_PRU_/CDP	Ratio (*w*/*w*) ABPBI/CDP	ABPBI, g (mol)	CsOH·H_2_O, g (mol)	PA Added, g (mol)	Elemental Analysis Cs—calc./Found P—calc./Found
**1**	1:0.8	39:61	1.16 (0.01)	1.344 (0.008)	3.72 (0.038)	C_7_H_14.6_Cs_0.8_N_2_O_15.2_P_3.8_ Cs—17.90/18.35 P—19.81/19.77
**2**	1:1	33:67	1.16 (0.01)	1.680 (0.01)	3.92 (0.04)	C_7_H_15_CsN_2_O_16_P_4_ Cs—20.77/20.25 P—19.36/18.27
**3**	1:1.2	30:70	1.16 (0.01)	2.015 (0.012)	4.12 (0.042)	C_7_H_15.4_Cs_1.2_N_2_O_16.8_P_4.2_ Cs—23.25/22.85 P—18.96/18.07

## Data Availability

The authors confirm that the data supporting the findings of this study are available within the article and its Supplementary Materials.

## Supplementary Materials

The following supporting information can be downloaded at: https://www.mdpi.com/article/10.3390/membranes16060203/s1, Figure S1: Formation of a preliminary film. The ABPBI/CsOH film on a MemCast Porometer NV setup; Figure S2: The ABPBI_PRU_·3PA/CDP membrane after phosphoric acid doping; Figure S3: SEM images of the ABPBI_PRU_·3PA/CDP membrane (ABPBI/CDP 1:1 mol/mol) at lower (a) and higher (b) magnification; Figure S4: (a) HAADF STEM image and the corresponding elemental maps for (b) general overlay, (c) C, (d) O, (e) P and (f) Cs; (g) EDX analysis of ABPBI_PRU_·3PA/CDP (1:1 mol/mol); Figure S5: EIS Nyquist plots for MEA1 at 160 °C (green), 180 °C (red), 200 °C (blue), 220 °C (orange) and 240 °C (yellow); Figure S6: EIS Nyquist plots for MEA 2 at 160 °C (green), 180 °C (orange) and 250 °C (yellow); Figure S7: EIS Nyquist plots for MEA 3 at 160 °C (green) and 180 °C (red); Figure S8: Polarization and power density curves for MEA 1 at 180 °C (yellow), 200 °C (blue), 220 °C (red) and 240 °C (green); Figure S9: MEA 2 after 1 week of operation at 180 °C: polarization and power density curves (top) and EIS Nyquist plot (bottom); electrode working area is 5 cm^2^; Figure S10: Polarization and power density curves for MEA 3 at 160 C (green), 180 C (yellow), 200 C (blue); Figure S11: Membrane resistance values from EIS Nyquist plots for MEA 1 (blue), MEA 2 (red) and MEA 3 (green); Figure S12: Maximum power density values from EIS Nyquist plots for MEA 1 (blue), MEA 2 (red) and MEA 3 (green); Figure S13: Oxidation current of hydrogen crossover through the membrane at 160 °C for H_2_/Ar operation (yellow) and LSV with short-circuit correction (green); Figure S14: Proton conductivity of the ABPBI membranes with ABPBI_PRU_/CDP ratios of 1:0.8 mol/mol (sample **1**, orange) and 1:1 mol/mol (sample **2**, green); Figure S15: Assumed proton transfer channels for ABPBI/CDP interphase boundary when doped with PA; Table S1: The eZAF Quant results for C, O, P and Cs.
