# Supplementary material for: Proton-Conducting Composite of Poly(2,5-benzimidazole) and Cesium Dihydrogen Phosphate—The Emerging of Ultrahigh-Temperature Polymer-Electrolyte Membrane Fuel Cell (UT-PEMFC)
Source: Membranes (Basel). 2026 Jun 10;16(6):203. doi: 10.3390/membranes16060203 (PMC13303277; doi:10.3390/membranes16060203)
Supplement: Supplementary file 1 [file membranes-16-00203-s001.zip › membranes-4190591-supplementary.pdf]

Supporting Information

Proton Conducting Composite of Poly(2,5-benzimidazole) and Cesium Dihydrogen Phosphate – the Emerging of Ultrahigh-Temperature Polymer-Electrolyte Membrane Fuel Cell (UT-PEMFC)

Kirill M. Skupov,[1*] Igor I. Ponomarev,[1] Elizaveta S. Vtyurina,[1] Alexey A. Bugerya,[1,2] Olga M. Zhigalina[3,4], Yulia A. Volkova,[1] Anna A. Lysova,[5] Yury A. Dobrovolsky[2]

[1] A.N. Nesmeyanov Institute of Organoelement Compounds of Russian Academy of Sciences, 28 Vavilova St., bld. 1, Moscow, 119334 Russia

[2] Hydrogen Energy Center LLC, 3 Akademika Semenova St., bld. 3., Chernogolovka, 142432 Russia

[3] Department of Materials Sciences, Bauman Moscow State Technical University, 5 2nd Baumanskaya St., Moscow, 105005 Russia

[4] A.V. Shubnikov Institute of Crystallography, Kurchatov Complex of Crystallography and Photonics, National Research Centre "Kurchatov Institute", 59 Leninsky Ave., Moscow 119333, Russia

[5] Kurnakov Institute of General and Inorganic Chemistry of the Russian Academy of Sciences, 31 Leninsky Ave., Moscow 119071, Russia

* Correspondence: kskupov@ineos.ac.ru (K.M.S.); gagapon@ineos.ac.ru (I.I.P.)

Table of Contents

1. Film Images……………………………………………………………………………3

2. Additional scanning electron microscopy data……………………………………..………4

3. HAADF STEM image, the corresponding elemental maps (for C, O, P and Cs) and EDX analysis of ABPBI$_{PRU}$·3PA/CDP (1:1 mol/mol)………………………………………………5

4. Additional electrochemical impedance spectroscopy (EIS) data…………………….…….12

5. Additional polarization and power density curves……………………………………….13

6. Membrane resistance and maximal power density data for different MEAs…………..…15

7. Linear sweep voltammetry data……………………………………………………….…….16

8. Proton conductivity of the membranes……………………………………………………...17

9. Assumed proton transfer channels for ABPBI/CDP interphase boundary………………..18

1. Film images

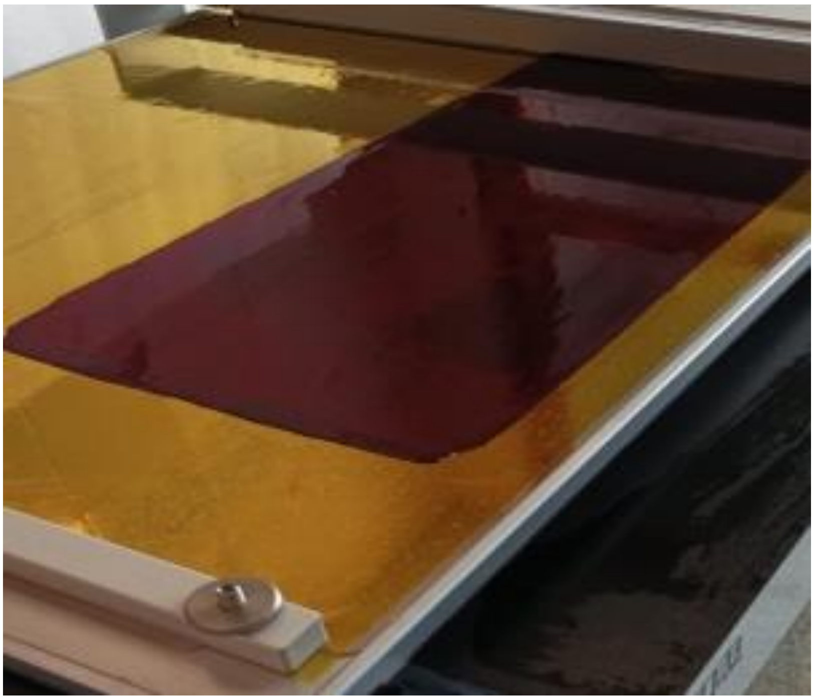

Figure S1. Formation of a preliminary film. The ABPBI/CsOH film on a MemCast Porometer NV setup.

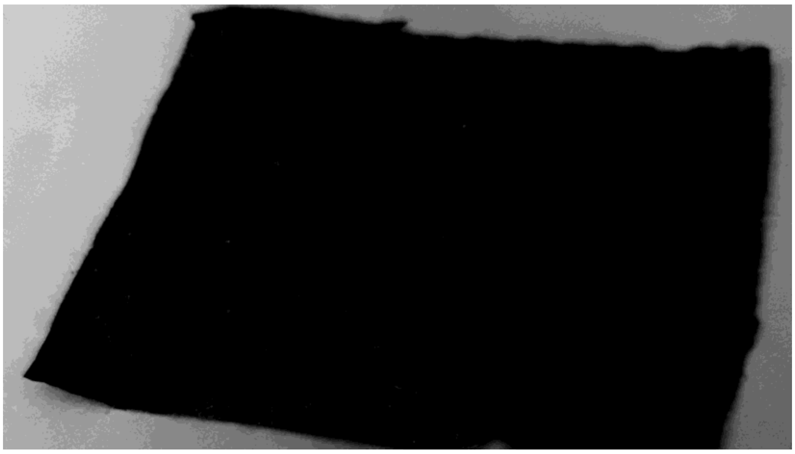

Figure S2. The $ABPBI_{PRU}$·3PA/CDP membrane after phosphoric acid doping.

2. Additional scanning electron microscopy data

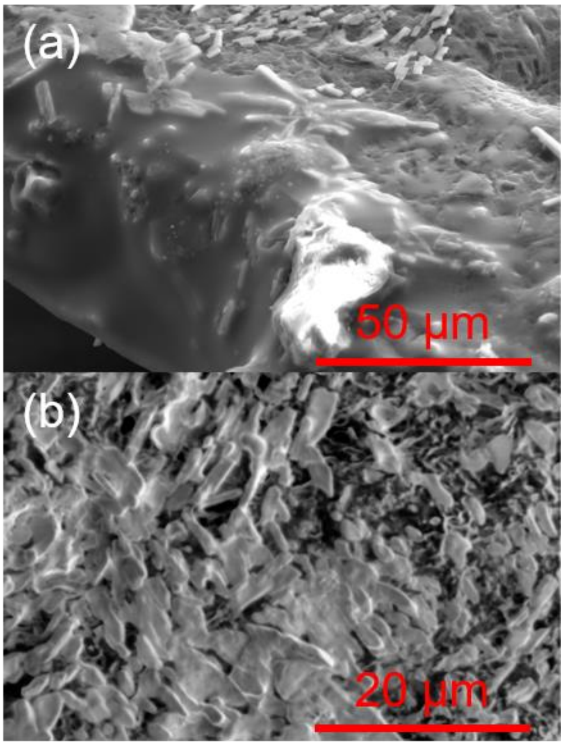

Figure S3. SEM images of the $ABPBI_{PRU}$·3PA/CDP membrane (ABPBI/CDP 1:1 mol/mol) at lower (a) and higher (b) magnification.

3. HAADF STEM image, the corresponding elemental maps (for C, O, P and Cs) and EDX analysis of ABPBIPRU·3PA/CDP (1:1 mol/mol)

(a)

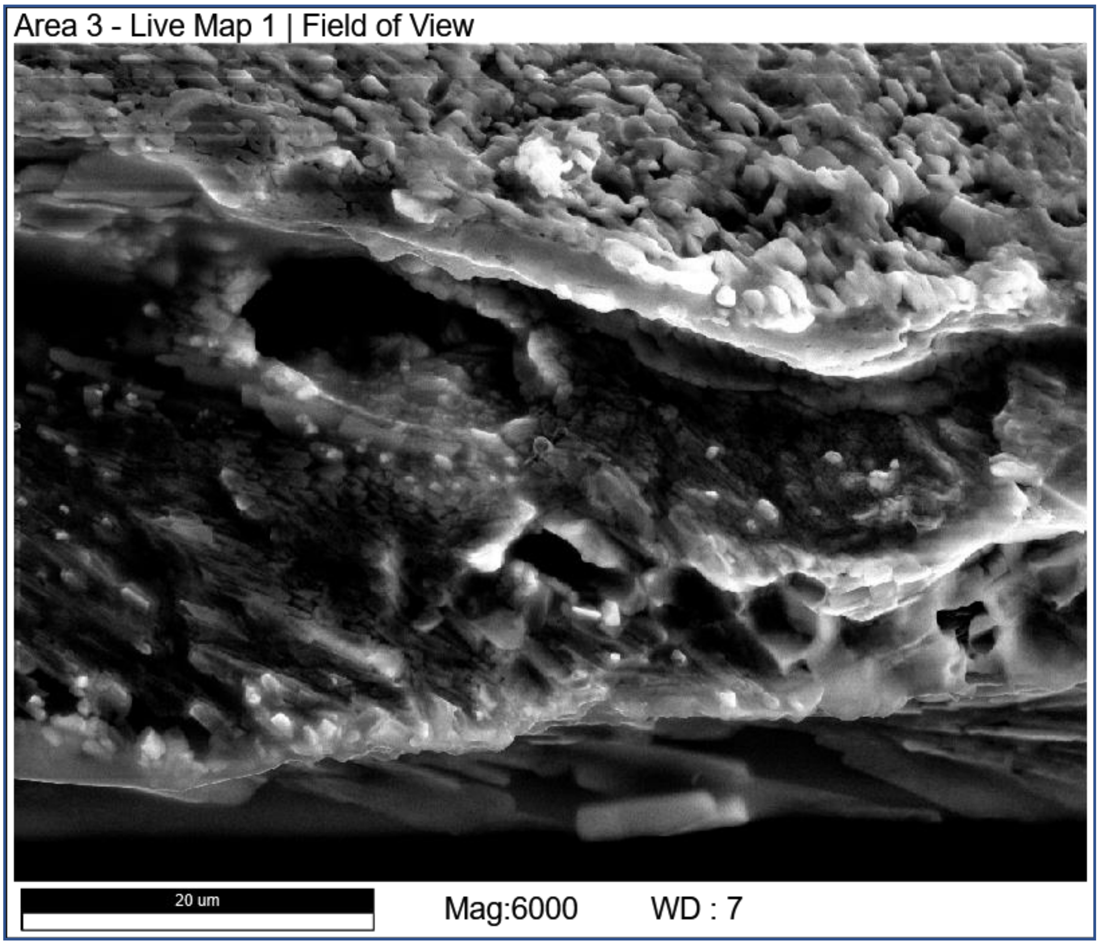

(b)

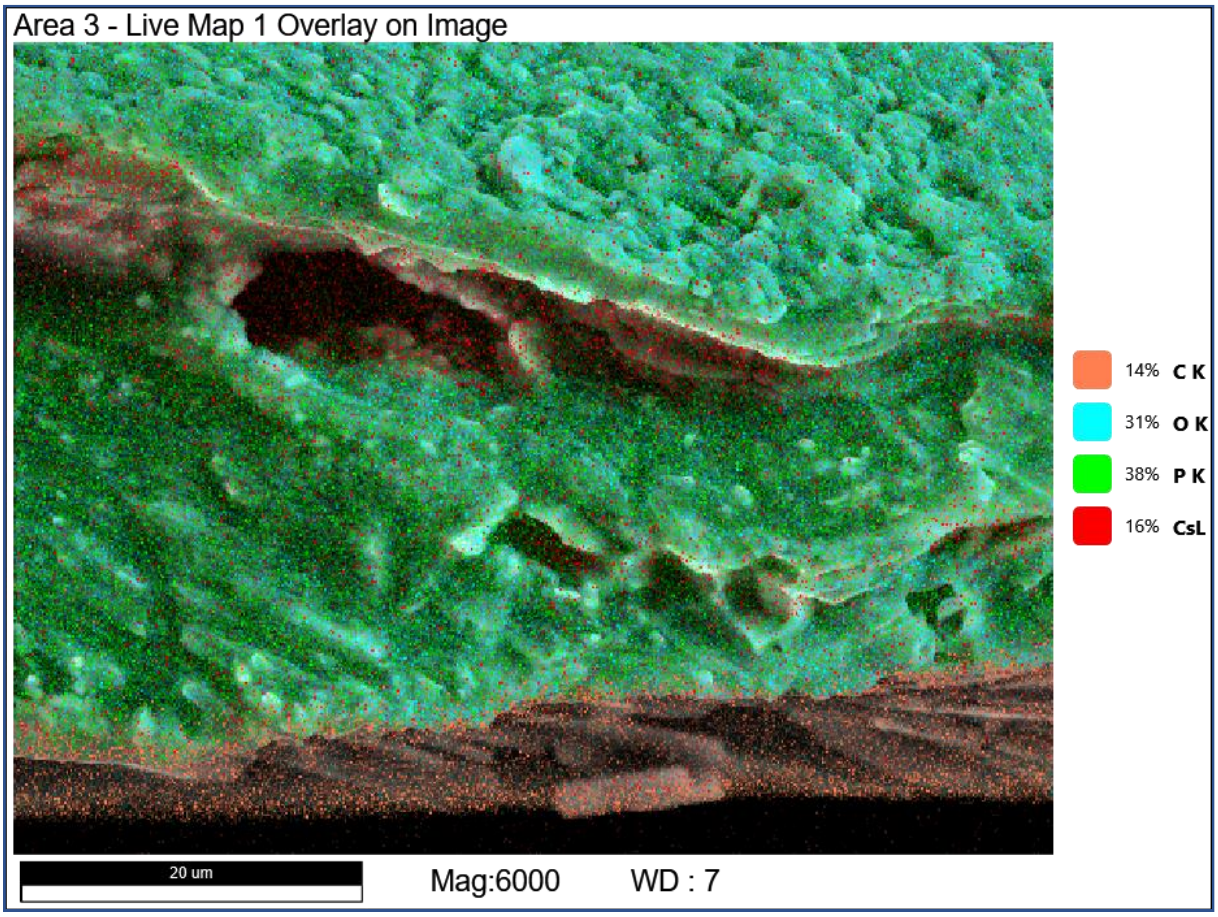

Area 3 - Live Map 1 Overlay on Image
14% C K
31% O K
38% P K
16% CsL
20 um
Mag:6000
WD : 7

(c)

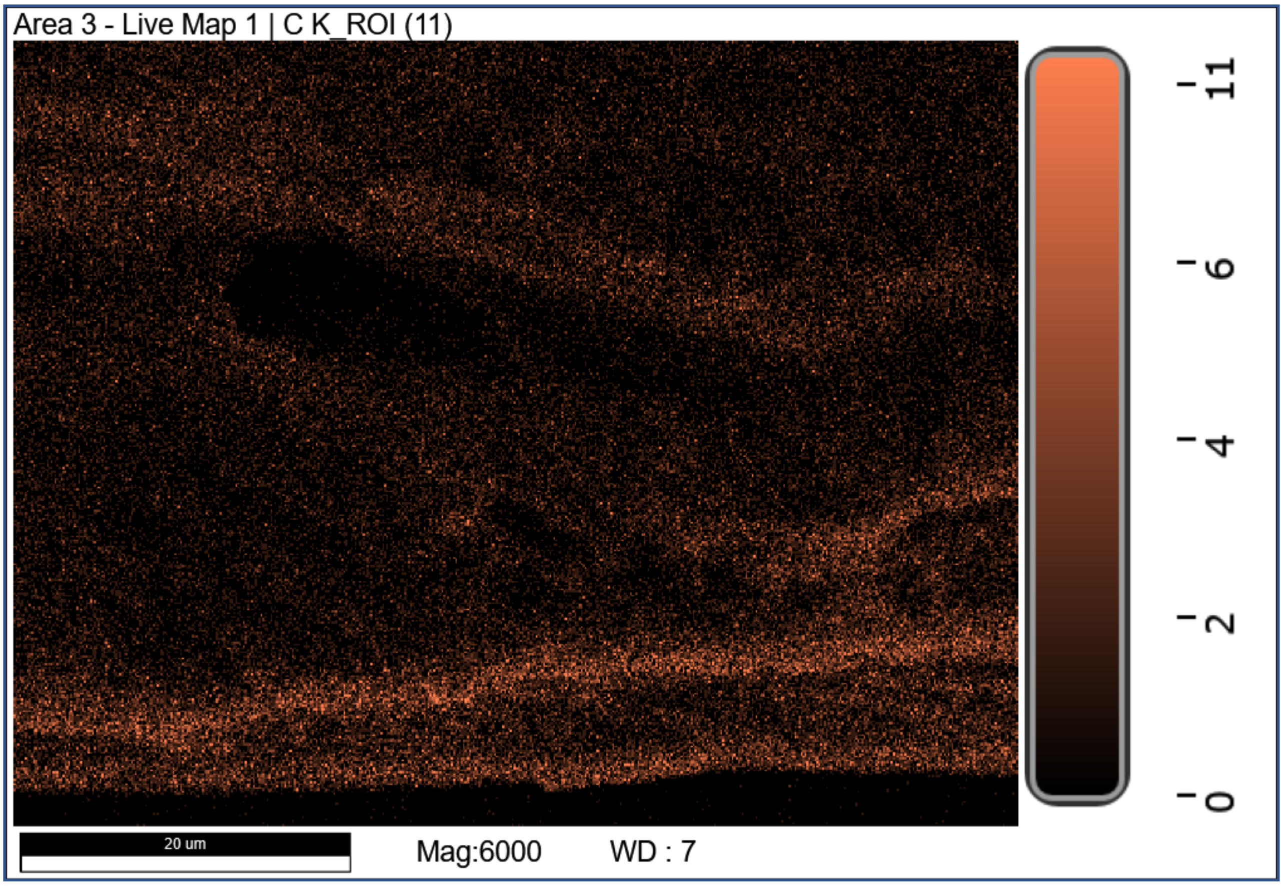

(d)

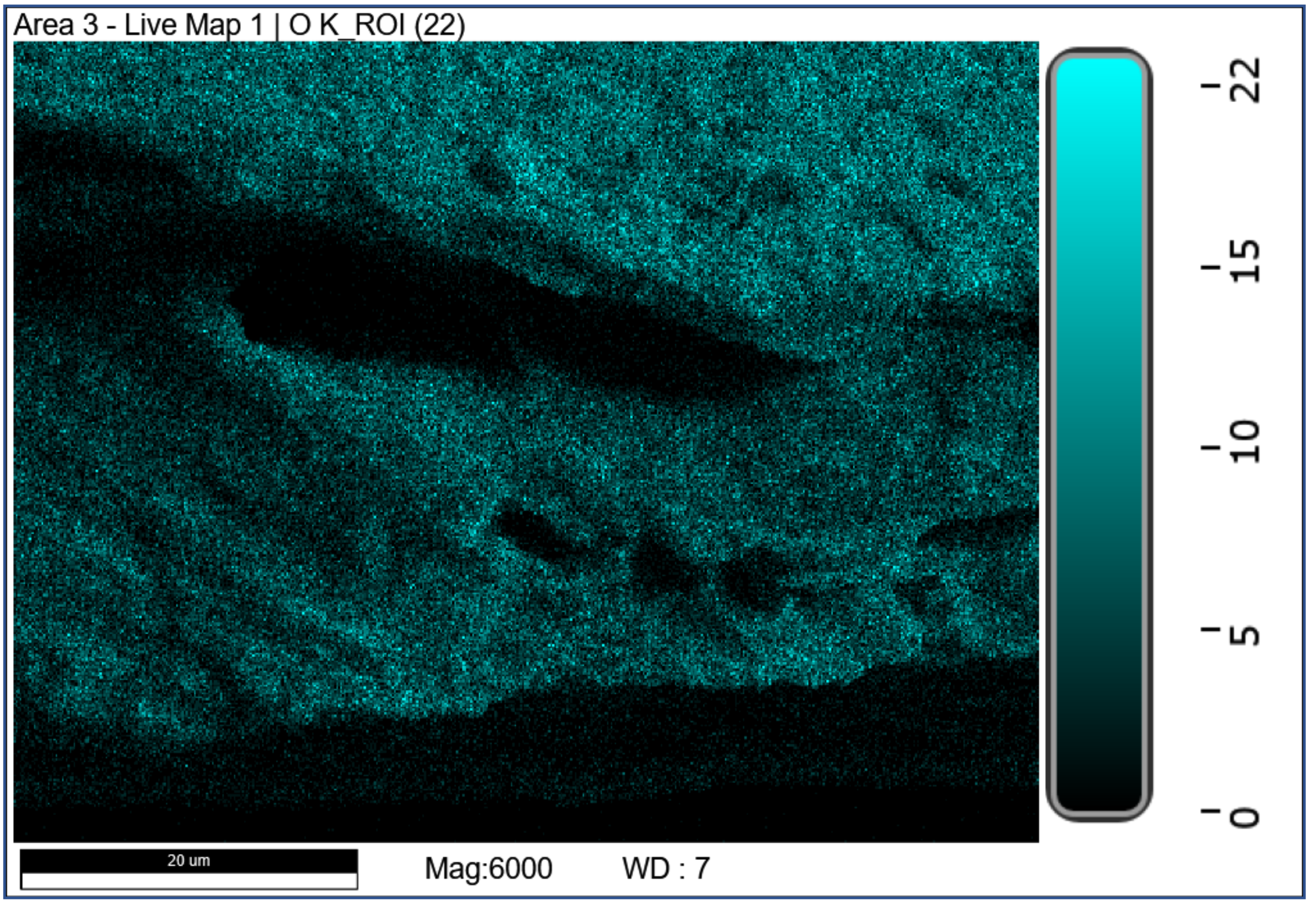

(e)

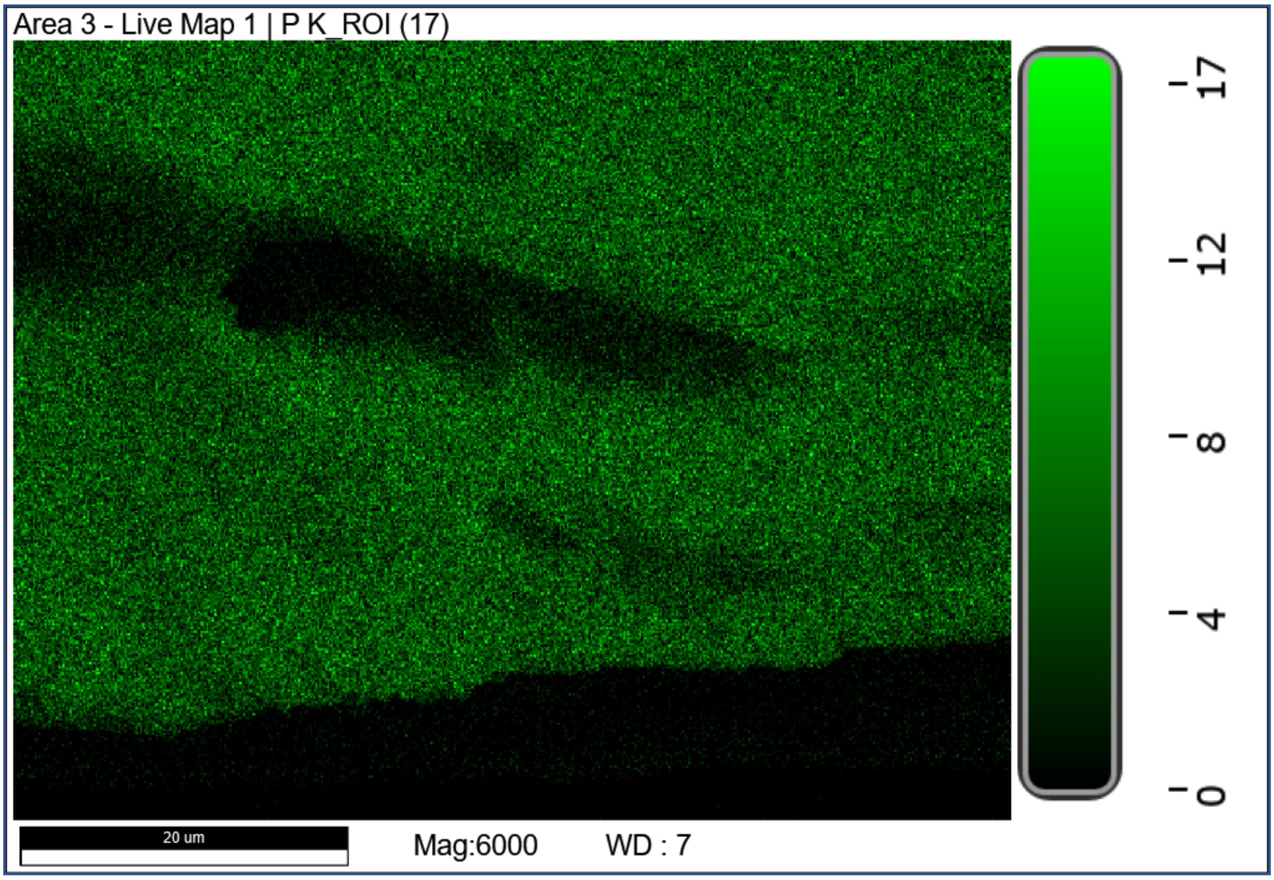

(f)

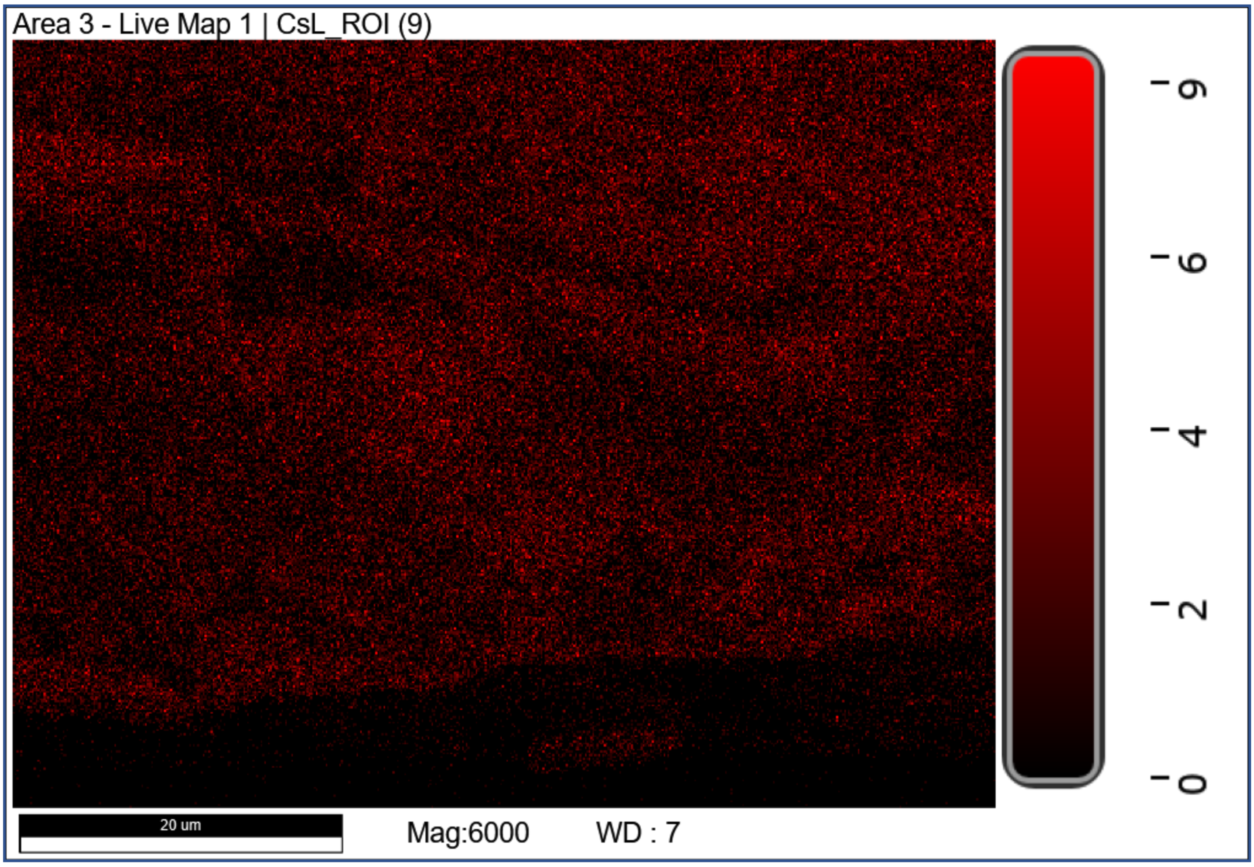

(g)

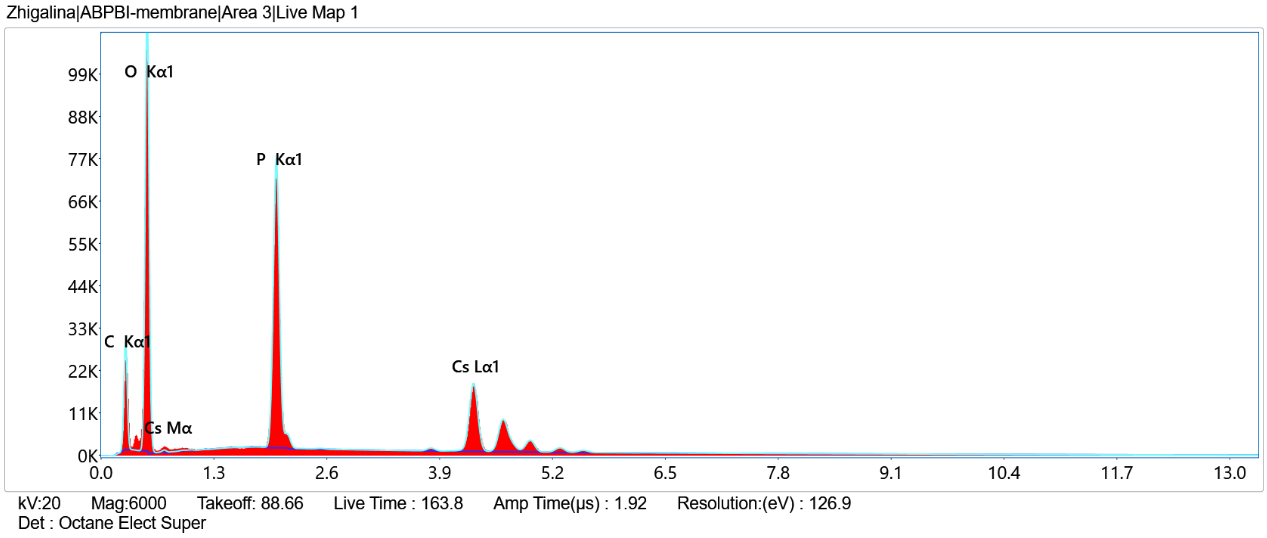

Figure S4. (a) HAADF STEM image and the corresponding elemental maps for (b) general overlay, (c) C, (d) O, (e) P and (f) Cs; and (g) EDX analysis of $ABPBI_{PRU}$·3PA/CDP (1:1 mol/mol).

Table S1. The eZAF Quant Results for C, O, P and Cs.

eZAF Quant Result - Analysis Uncertainty: 10.47 %

Element	Weight %	MDL	Atomic %	Error %	Net Int.	R	A	F
C K	34.4	0.18	49.2	10.8	801.8	0.8906	0.0527	1.0000
O K	39.5	0.03	42.5	10.2	3858.5	0.9022	0.0854	1.0000
P K	11.6	0.02	6.4	4.3	3898.5	0.9307	0.6889	1.0094
Cs L	14.5	0.08	1.9	2.2	1264.1	0.9530	0.9399	1.0084

4. Additional electrochemical impedance spectroscopy (EIS) data

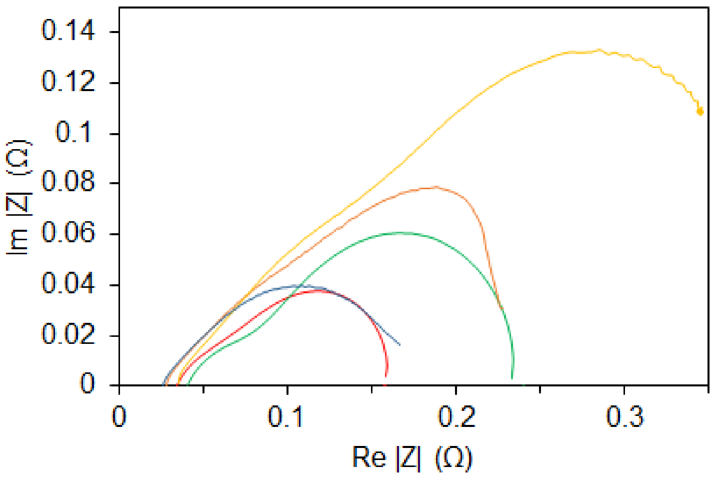

Figure S5. EIS Nyquist plots for MEA1 at 160 °C (green), 180 °C (red), 200 °C (blue), 220 °C (orange) and 240 °C (yellow).

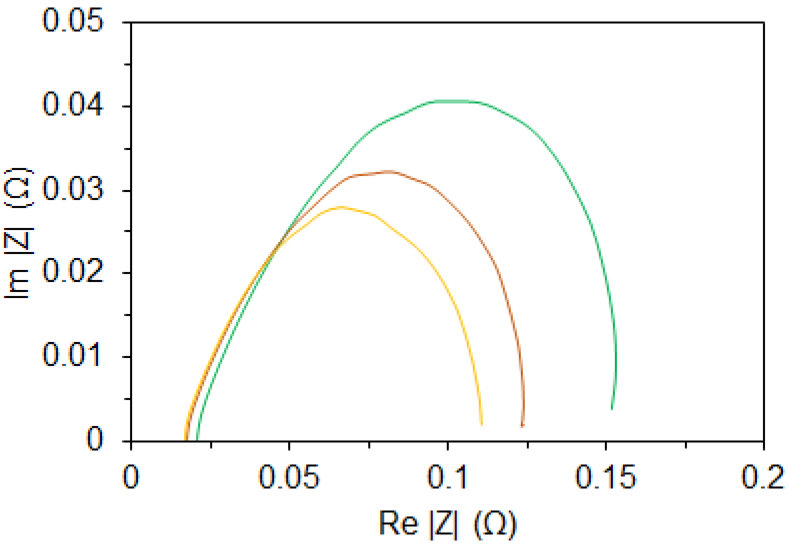

Figure S6. EIS Nyquist plots for MEA 2 at 160 °C (green), 180 °C (orange) and 250 °C (yellow).

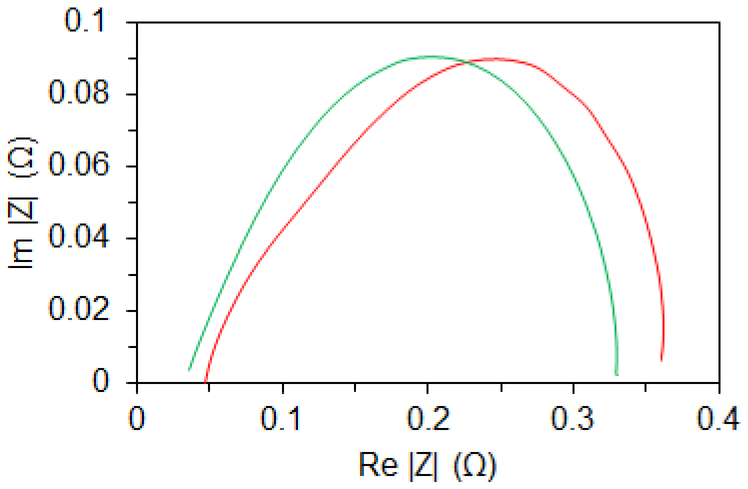

Figure S7. EIS Nyquist plots for MEA 3 at 160 °C (green) and 180 °C (red).

5. Additional polarization and power density curves

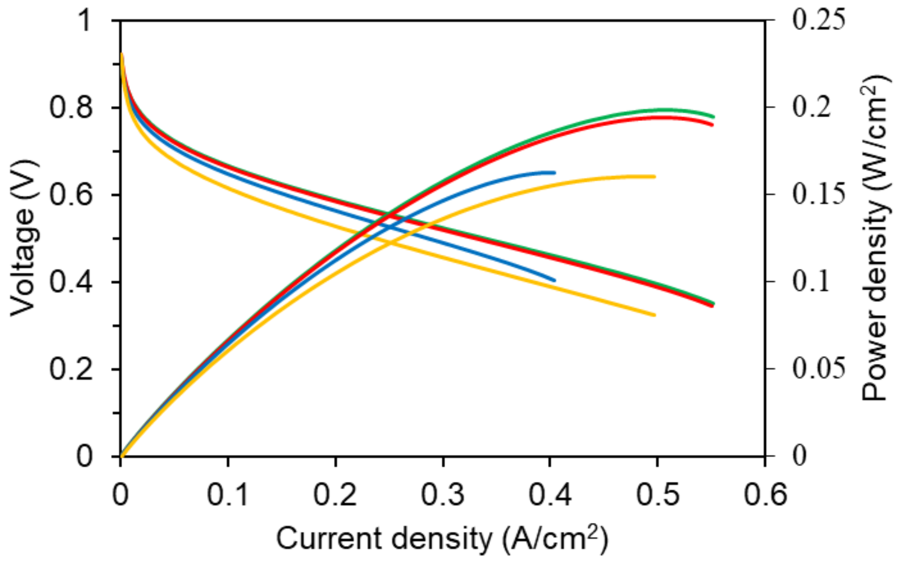

Figure S8. Polarization and power density curves for MEA 1 at 180 °C (yellow), 200 °C (blue), 220 °C (red) and 240 °C (green).

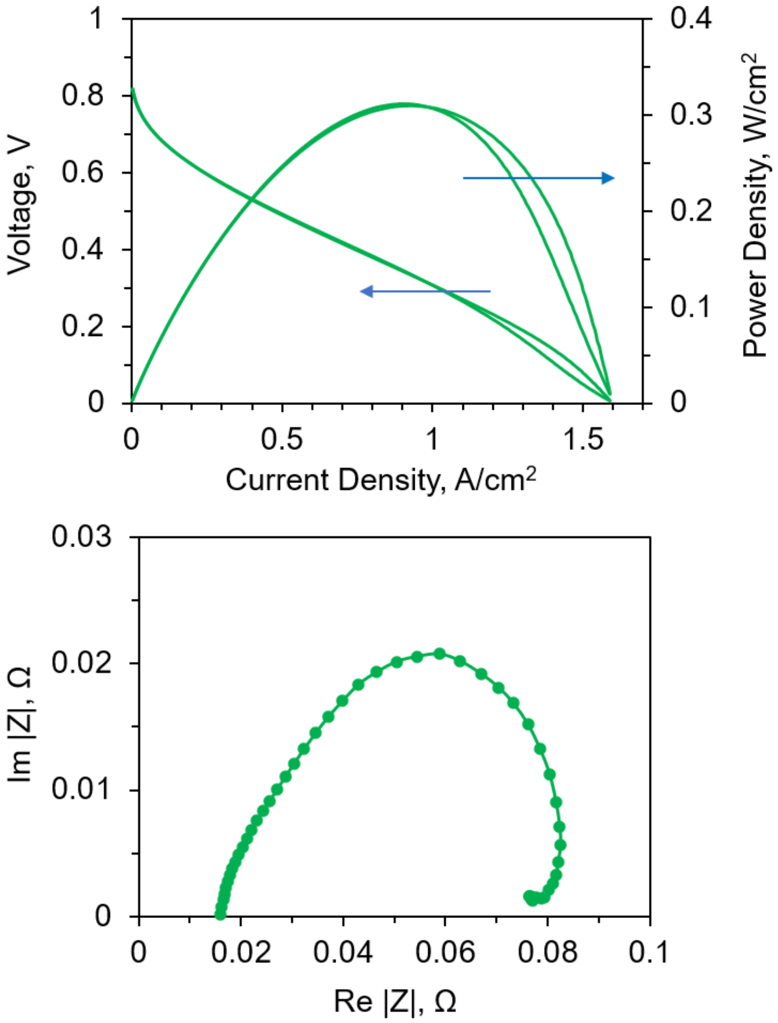

Figure S9. MEA 2 after 1 week of operation at 180 °C: polarization and power density curves (top), EIS Nyquist plot (bottom), electrode working area is 5 cm^2.

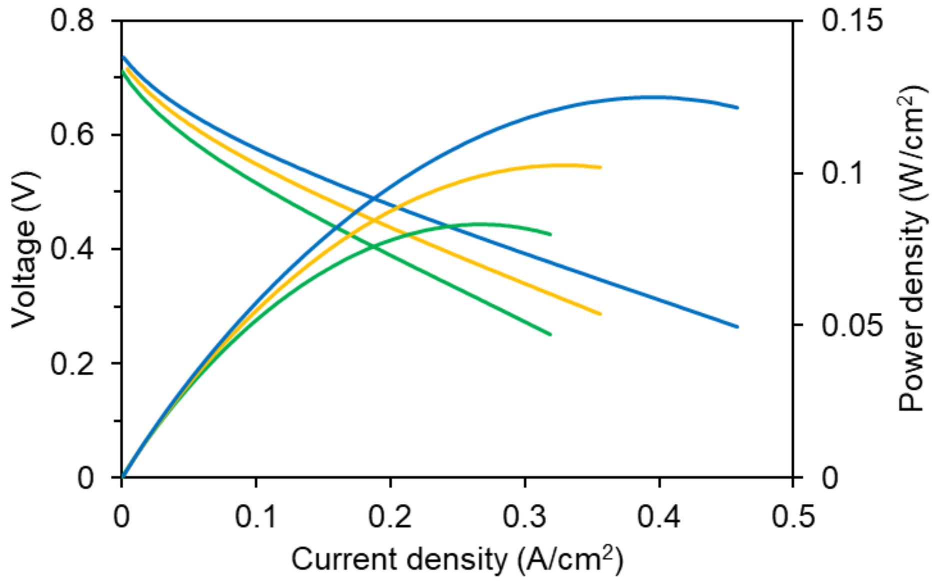

Figure S10. Polarization and power density curves for MEA 3 at 160 C (green), 180 C (yellow), 200 C (blue).

6. Membrane resistance and maximal power density data for different MEAs

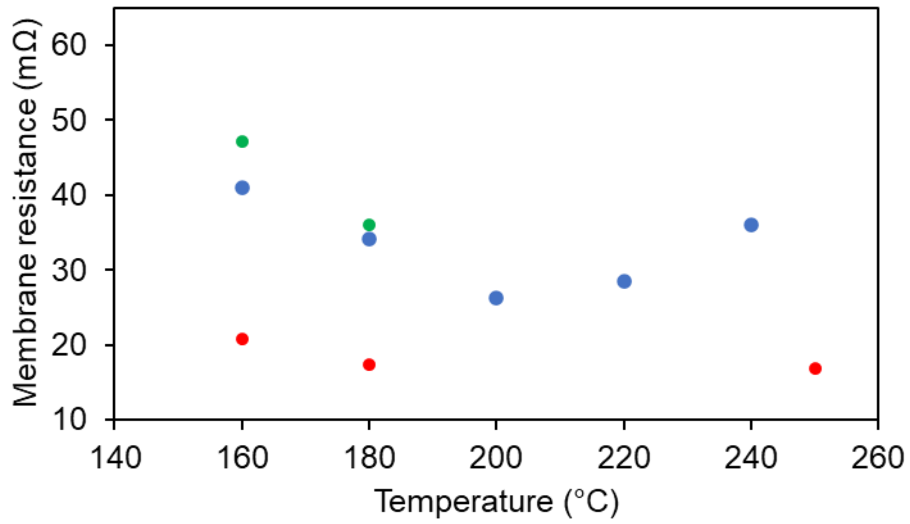

Figure S11. Membrane resistance values from EIS Nyquist plots for MEA 1 (blue), MEA 2 (red) and MEA 3 (green).

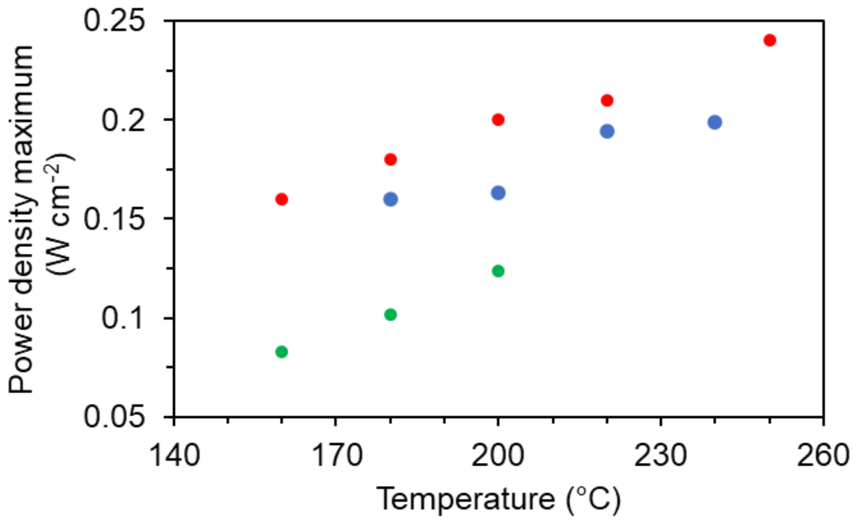

Figure S12. Maximum power density values from EIS Nyquist plots for MEA 1 (blue), MEA 2 (red) and MEA 3 (green).

7. Linear sweep voltammetry

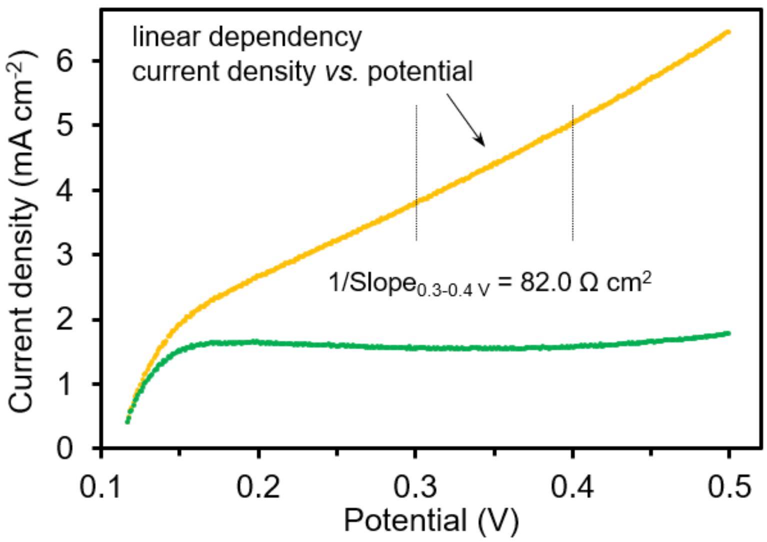

Figure S13. Oxidation current of hydrogen crossover through the membrane at 160 °C for H_2/Ar operation (yellow) and LSV with short-circuit correction (green).

8. Proton conductivity of the membranes

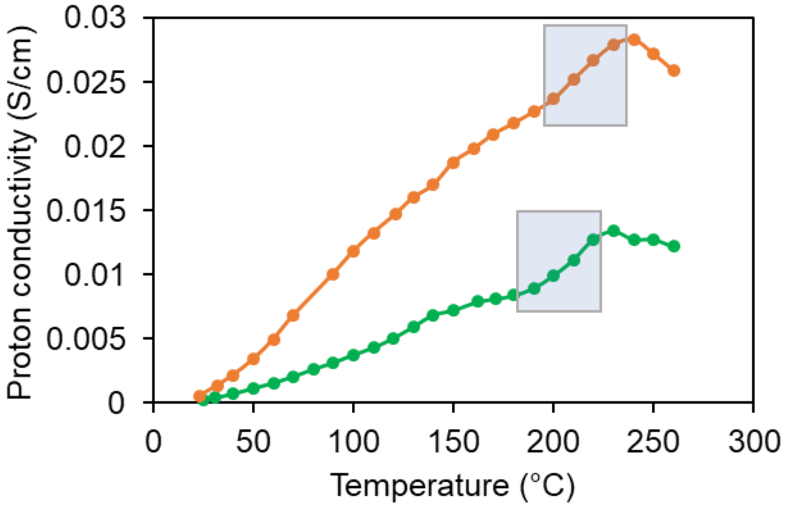

Figure S14. Proton conductivity of the ABPBI membranes with $ABPBI_{PRU}$/CDP ratios of 1:0.8 mol/mol (sample **1**, orange) and 1:1 mol/mol (sample **2**, green).

9. Assumed proton transfer channels for ABPBI/CDP interphase boundary

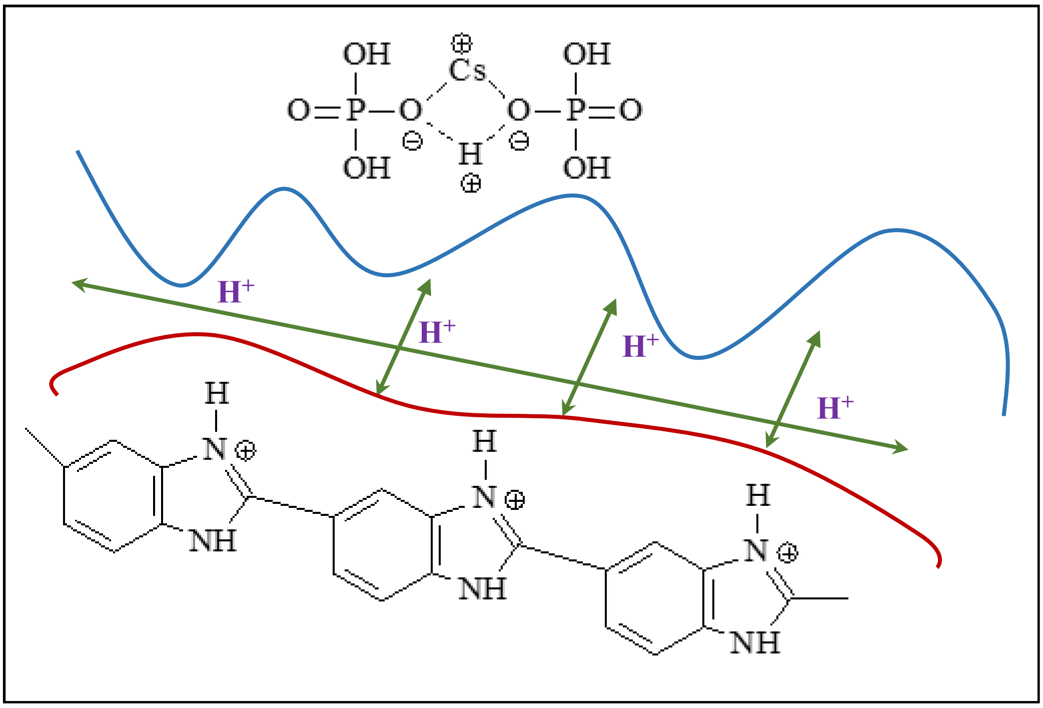

Figure S15. Assumed proton transfer channels for ABPBI/CDP interphase boundary when doped by PA.
